# ﻿Molecular analysis of *Lepidopleuruscajetanus* (Poli, 1791) (Polyplacophora, Leptochitonidae) from the Mediterranean and near Atlantic

**DOI:** 10.3897/zookeys.1099.75837

**Published:** 2022-05-03

**Authors:** Mariastella Colomba, Julia D. Sigwart, Walter Renda, Armando Gregorini, Maurizio Sosso, Bruno Dell’Angelo

**Affiliations:** 1 University of Urbino, Dept. of Biomolecular Sciences, via I. Maggetti 22, 61029 Urbino (PU), Italy; 2 Senckenberg Research Institute and Museum, Senckenberganlage 25, 60325 Frankfurt, Germany; 3 Via Bologna 18/A, 87032 Amantea (CS), Italy; 4 Via Bengasi 4, 16153 Genova (GE), Italy; 5 Via Briscata 16, 16154 Genova (GE), Italy

**Keywords:** 16S rRNA, chitons, COI, phylogeny, standard mitochondrial markers

## Abstract

In the present paper we used a molecular data set (including mitochondrial partial 16S rRNA and COI gene sequences) to examine the genetic structure of *Lepidopleuruscajetanus* (Poli, 1791) (Polyplacophora, Leptochitonidae) - a distinctive shallow water chiton and member of the basal branching Lepidopleurida, which is widespread in and adjacent to the Mediterranean. The analyses of the two mt-standard marker fragments resolved two main discrete clusters reported as *L.cajetanus* s.s. and L.aff.cajetanus, respectively. *Lepidopleuruscajetanus* s.s. is widespread throughout the area under study, while the second distinct lineage apparently co-occurs on the eastern Spanish mainland coast of the Balearic Sea. This result is discussed comparing our data with those reported, in 2014, by Fernández and colleagues who described *L.cajetanus* as exhibiting “a ‘chaotic patchiness’ pattern defined by a high genetic variability with locality-exclusive haplotypes, high genetic divergence, and a lack of geographic structure”. Although genetic data alone are not sufficient to draw any definitive conclusions, nevertheless we believe that present results shed new light on *L.cajetanus* which apparently shows more geographically patterned genetic structure than supposed so far.

## ﻿Introduction

Chitons (class Polyplacophora) are the third-largest class in the phylum Mollusca by species richness of living taxa (Ponder et al. 2020). The superficial similarity of the living species, with a distinctive eight-part shell armour covering a soft foot that adheres to the substratum, has created some long-term confusion in the ecological identification of species. Yet *Lepidopleuruscajetanus* (Poli, 1791) (Polyplacophora, Leptochitonidae) was recognised as a distinct form in the Mediterranean very early in the history of formal modern taxonomy. Although it is phylogenetically nested within the genus *Leptochiton* s.s. with other species in this clade from the Mediterranean and North Atlantic (Sigwart et al. 2011), taxonomists have maintained the genus *Lepidopleurus* in acknowledgement of its unique morphology. WoRMS (World Register of Marine Species) reports two living species of *Lepidopleurus*, *L.cajetanus* (Poli, 1791) and *L.cullierti* Roch, 1891 (https://www.marinespecies.org/aphia.php?p=taxdetails&id=138116). Actually, Castellanos (1988) cites *L.cullierti* showing also a figure of it (later taken up by Forcelli 2000), without taking into account that this species was already considered by Pilsbry (1893: 111) a *nomen dubium* (certainly not a *Lepidopleurus*, but belonging probably to the genus *Chaetopleura*). The species is not reported in recent papers dealing with Leptochitonidae from Chile (Sirenko 2006; Schwabe 2009; Schwabe and Sellanes 2010; Sirenko 2015; Sirenko and Sellanes 2016), but it is still (erroneously) reported in various lists. Recently, Aldea et al. (2020) reported *L.cullierti* as a dubious species. In conclusion, at least as far as concerns living species, *Lepidopleurus* is presently a monotypic genus. Notably, nomenclature is still confusing and a clear distinction between *Lepidopleurus* and *Leptochiton* has not been fully achieved.

*Lepidopleurus* was the first genus name proposed for lepidopleuran chitons, including only the species *L.cajetanus*. In 1847, Gray established the genus name *Leptochiton*. Both genera were included in the family Leptochitonidae Dall, 1889 with *Leptochitonasellus* as the type species. A few years later, Pilsbry (1892) listed *Leptochiton* as a junior subjective synonym of *Lepidopleurus*, and changed the family name to Lepidopleuridae. Since then, *Lepidopleurus* and *Leptochiton* (and family names) have been used more or less interchangeably (Sigwart et al. 2011). To date, there is insufficient evidence to separate *Lepidopleurus* and *Leptochiton* s.s. as distinct genera, even if they are often distinguished on the basis of shell thickness and sculpture [i.e., distinctive shell morphology with pronounced concentric ridges on the lateral areas and terminal valves (*Lepidopleurus*), or flat and plain shells generally lacking strong raised sculpture (*Leptochiton*)].

*Lepidopleuruscajetanus* is widespread throughout the Mediterranean (where, even if quite discontinuously, it can be very common locally) and more rarely in the Atlantic, from the Iberian Peninsula (Spain and Portugal) to Morocco and to the Canary Islands (Spain) and Berlengas Archipelago (Portugal) (Kaas and Van Belle 1985; Dell’Angelo and Smriglio 2001). The species is known as fossils in the European Neogene: from the lower Miocene (Burdigalian) of the Aquitaine Basin to the middle Miocene of the Aquitaine Basin and Paratethys and the French and Italian upper Miocene, to the Pliocene of Italy, Spain and Greece, to the Pleistocene of Italy and Greece (Dell’Angelo et al. 2018a, 2018b and references therein). As has been widely described (see for example, Dell’Angelo et al. 2013, 2015, 2018a, 2018b), fossils of *L.cajetanus* show great variability in the morphological characters of the plates (sculpture, shape, etc.), which is much less evident in the living specimens.

Living chitons are broadly divided into two main clades, the orders Lepidopleurida and Chitonida. The former group retains plesiomorphic shell forms and is therefore particularly interesting for studies of molluscan phylogeny (Sigwart et al. 2011). Most members of Lepidopleurida inhabit deep sea environments, but as *Lepidopleuruscajetanus* can be found intertidally it has been widely used in genetic studies including molecular phylogenetic studies of molluscs (see for example, Giribet and Wheeler 2002).

In addition to data on the impact of strong biogeographical barriers on gene flow (Ayre et al. 2009), other studies on chiton population genetics have recovered well-mixed populations in spite of geographic barriers (e.g., Doonan et al. 2012), including some species in the Mediterranean such as *Rhyssoplaxolivacea* (Spengler, 1797) (Fernández et al. 2014). One study described a cryptic species on the basis of differential haplotype structures which were attributed to potentially different dispersal capacity of two species of *Leptochiton* s.l. (Sigwart and Chen 2018): *L.rugatus* (Carpenter in Pilsbry, 1892) and *L.cascadiensis* Sigwart & Chen, 2018. In contrast to previous results for chitons, a focussed study on the population genetic structure of *Lepidopleuruscajetanus* from the Atlantic and Mediterranean coasts found ‘chaotic patchiness’ defined by unique haplotypes, high genetic divergence, and yet no apparent geographic partitioning (Fernández et al. 2014). In particular, the authors found two major clades, one of which was divided into two subclades. The possibility that some of these lineages represented multiple cryptic species in *L.cajetanus* was raised but, eventually, dismissed “because of the unique morphology of *L.cajetanus*”. Starting from that paper, in the present study, we collected genetic samples from additional locations, expanding the geographical coverage examined for *Lepidopleuruscajetanus*, in order to test whether increasing the number of samples and adding new collection sites could confirm the pattern already described as suggesting old and stable populations with, however, limited distinguishable geographical structure. Alternatively, by filling in more of the geographic range of this species, new results could help resolve a broader structure of distinctive co-occurring but separate clades.

## ﻿Materials and methods

Thirteen (13) *Lepidopleuruscajetanus* specimens were sampled from the Atlantic and Mediterranean coasts of Spain, Italy, Croatia and the Canary Islands by two of the authors (BDA and WR) and other collectors (Table 1).

**Table 1.** GenBank accession numbers of 16S rRNA and COI partial sequences of the specimens used in the study and reported in the phylogenetic tree.

Species / sample nr	COI	16S rRNA	Collection site (CS)	CS nr	Reference
***L.cajetanus* s.s.**					
1	KF052983	KF052732	Cadaques (Girona, Spain)	2	b
3	KF052981	KF052735	Cadaques (Girona, Spain)	2	b
4	KF052980	KF052737	Cadaques (Girona, Spain)	2	b
5	KF052979	KF052713	Tossa de Mar (Girona, Spain)	3	b
6	KF052978	KF052724	Tossa de Mar (Girona, Spain)	3	b
7	KF052977	KF052715	Tossa de Mar (Girona, Spain)	3	b
8	KF052976	KF052723	Tossa de Mar (Girona, Spain)	3	b
9	KF052975	KF052725	Tossa de Mar (Girona, Spain)	3	b
10	KF052974	KF052729	Tossa de Mar (Girona, Spain)	3	b
12	KF052972	KF052728	Calafat (Tarragona, Spain)	9	b
13	KF052971	KF052711	Calafat (Tarragona, Spain)	9	b
14	KF052970	KF052733	Cabo de Palos (Murcia, Spain)	1	b
15	KF052969	KF052714	Cabo de Palos (Murcia, Spain)	1	b
16	KF052968	KF052731	Mar Menuda, Tossa de Mar (Girona, Spain)	10	b
17	KF052967	KF052738	Mar Menuda, Tossa de Mar (Girona, Spain)	10	b
18	KF052966	KF052730	Mar Menuda, Tossa de Mar (Girona, Spain)	10	b
19	KF052965	KF052734	Mar Menuda, Tossa de Mar (Girona, Spain)	10	b
23	KF052960	KF052727	Mar Menuda, Tossa de Mar (Girona, Spain)	10	b
24	KF052959	KF052726	Mar Menuda, Tossa de Mar (Girona, Spain)	10	b
26	KF052957	KF052721	Cadaques (Girona, Spain)	2	b
27	KF052956	KF052722	Xabia (Alicante, Spain)	5	b
28	KF052954	KF052736	Xabia (Alicante, Spain)	5	b
30	KF052952	KF052712	NA	NA	b
31	KF052951	KF052719	Rhodes (Greece)	7	b
32	KF052950	KF052720	Rhodes (Greece)	7	b
33	KF052948	KF052718	Rhodes (Greece)	7	b
34	KF052947	KF052717	Rhodes (Greece)	7	b
35	KJ500166	KJ500177	Santa Maria Navarrese (Sardinia, Italy)	23	b
36	AF120626	AY377585	NA	NA	a
37	KF052961		Mar Menuda, Tossa de Mar (Girona, Spain)	10	b
38	KF052955		Xabia (Alicante, Spain)	5	b
39	KF052949		Rhodes (Greece)	7	b
40	KF052944		Cabrera (Balearic Islands, Spain)	8	b
41	KF052945		Cabrera (Balearic Islands, Spain)	8	b
42	KF052946		Cabrera (Balearic Islands, Spain)	8	b
44		KF052709	Tossa de Mar (Girona, Spain)	3	b
46		KF052710	Rhodes (Greece)	7	b
47		KF052716	Rhodes (Greece)	7	b
**A**		MW748076	Torre Ovo (Taranto, Italy)	24	c
**B**	MW751980	MW748077	Chia, Cagliari (Sardinia, Italy)	23A	c
**C**		MW748078	Aguilas (Murcia, Spain)	1A	c
**D**	MW751981		Playa de Las Heras (Tenerife, Canary Is.)	26	c
**E**		MW748079	Arzachena, Sassari (Sardinia, Italy)	23A	c
**F**		MW748080	Tertenia, Nuoro (Sardinia, Italy)	23A	c
**G**	MW751982		Tertenia, Nuoro (Sardinia, Italy)	23A	c
**H**	MW751983	MW748081	Poetto, Cagliari (Sardinia, Italy)	23A	c
**I**	MW751984	MW748082	San Lucido (Cosenza, Italy)	24	c
**J**	MW751985	MW748083	Aguilas (Murcia, Spain)	1A	c
**K**	MW751986	MW748084	Umago (Croatia)	25	c
**L**		MW748085	Lussino Is. (Croatia)	25	c
**M**	MW751987		Vrsar, Orsera (Croatia)	25	c
**L.aff.cajetanus**					
2	KF052982	KF052702	Cadaques (Girona, Spain)	2	b
11	KF052973	KF052707	Tossa de Mar (Girona, Spain)	3	b
20	KF052964	KF052708	Mar Menuda, Tossa de Mar (Girona, Spain)	10	b
21	KF052963	KF052706	Mar Menuda, Tossa de Mar (Girona, Spain)	10	b
22	KF052962	KF052703	Mar Menuda, Tossa de Mar (Girona, Spain)	10	b
25	KF052958	KF052700	Cadaques (Girona, Spain)	2	b
29	KF052953	KF052705	Xabia (Alicante, Spain)	5	b
43		KF052701	Tossa de Mar (Girona, Spain)	3	b
45		KF052699	Cabo de Palos (Murcia, Spain)	1	b
***Rhyssoplaxolivaceus***					
1–16	KJ500158 – KJ500165, KF052941 – KF052942, KF052875 – KF052877, KF052885 – KF052887, KF052889	KJ500168 – KJ500174, KJ500176, KF052739 – KF052740, KF052778, KF052800 – KF052802, KF052791 – KF052792			
***Ischnochiton* spp.**					
	AY377704 – AY377709	AY377593 – AY377596			

*Lepidopleuruscajetanus* specimens are indicated by numbers (available data) or letters (present study) along with collection sites, collection site numbers and reference: a: Giribet and Wheeler (2002); b: Fernández et al. (2014); c: present study.

Total genomic DNA was isolated from a small piece of tissue taken from the foot of ethanol-preserved specimens. The extractions were carried out using the Wizard Genomic DNA Purification Kit (Promega). All the DNA extractions were kept at 4 °C for short-time use. Undiluted or different dilutions (from 1:10 to 1:50, based on the DNA concentration) of each DNA extraction were used as templates for PCR amplification of a portion of each of the two loci: the mitochondrial large subunit ribosomal DNA (mt-16S rRNA) and the cytochrome oxidase subunit I (mt-COI) genes. For the COI gene the primers used were LCO1490 (5’-GGTCAACAAATCATAAAGATATTGG-3’) and HCO2198 (5’-TAAACTTCAGGGTGACCAAAAAATCA-3’) (Folmer et al. 1994). PCR conditions involved an initial denaturation step at 95 °C for 5 min; then 35 cycles of denaturation at 95 °C for 1 min, annealing at 42 °C for 1 min and extension at 72 °C for 1 min; followed by a final extension step at 72 °C for 5 min. For the 16S rRNA gene, the primers used were 16sF (5’-CGGCCGCCTGTTTATCAAAAACAT-3’) and 16sR (5’-GGAGCTCCGGTTTGAACTCAGATC-3’) (Palumbi et al. 1991). The PCR conditions involved an initial denaturation step at 95 °C for 5 min; then 35 cycles of denaturation at 95 °C for 1 min, annealing at 50 °C for 1 min and extension at 72 °C for 1 min; followed by a final extension at 72 °C for 5 min. Amplified products were purified using the Wizard SV Gel and PCR CleanUp System (Promega).

*Pinnamuricata* Linnaeus, 1758 (Bivalvia) and *Haliotisdiscus* Reeve, 1846 (Gastropoda) were selected as outgroup for molecular analysis following the prior study by Fernández et al. (2014). *Pinnamuricata* and *H.discus* 16S rRNA and COI partial sequences (AB076929, GQ166570, AM049335 and AY146392), retrieved from GenBank, were added to homologous sequences of *Lepidopleuruscajetanus* previously studied (Giribet and Wheeler 2002; Fernández et al. 2014) and of *L.cajetanus* examined in the present study for the first time, with a total of 60 *L.cajetanus* ingroup terminals. Sixteen *Rhyssoplaxolivacea* (Spengler, 1797) and four *Ischnochiton* spp., were also added to the analysis (Table 1). All the sequences for each gene were aligned with BioEdit ClustalW. The substitution model for each partition was determined via the CIPRES Science Gateway (http://www.phylo.org/) (Miller et al. 2010) by the tool jModelTest of XSEDE. MrBayes analysis of multiple sequence alignment (COI+16S rRNA genes, in nexus format) was run on CIPRES by MrBayes on XSEDE, with the parameters for the consensus tree (50% majority rule, excluding 25% of trees as burnin) specified on the MrBayes block. All sequences generated in the present study were deposited in NCBI GenBank (Table 1).

Automatic Barcode Gap Discovery (ABGD) was also used on all available *L.cajetanus* COI sequence data (Puillandre et al. 2012) in order to tentatively delimit potential genetic lineages. Finally, Population Analysis with Reticulate Trees (PopART; Leigh and Bryant 2015) was employed to infer the *L.cajetanus* haplotype networks by the TCS (Templeton, Crandall and Sing) method.

## ﻿Results and discussion

**Figure 1.**
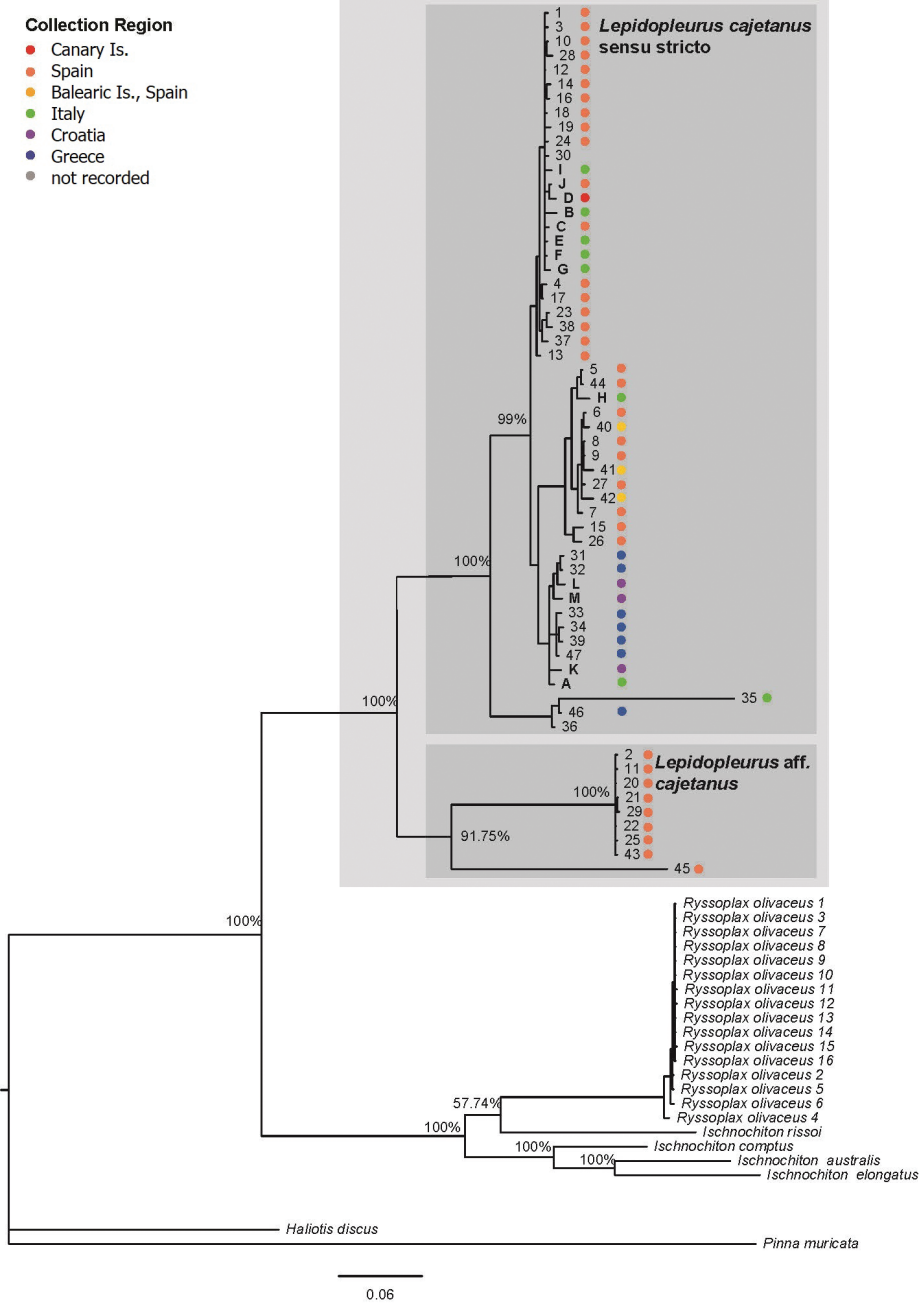
Bayesian phylogenetic tree obtained with MrBayes on the basis of a multiple sequence alignment (COI+16S rRNA genes) analysis. Nodal supports are Bayesian inference posterior probability (expressed in percentage). Scale bar represents units of length in expected substitutions per site. *Lepidopleuruscajetanus* specimens previously analysed (Giribet and Wheeler 2002; Fernández et al. 2014) are indicated by numbers, *L.cajetanus* specimens added in the present study are indicated by letters in bold. Colours correspond to the geographic distribution (see also Table 1).

**Figure 2.**
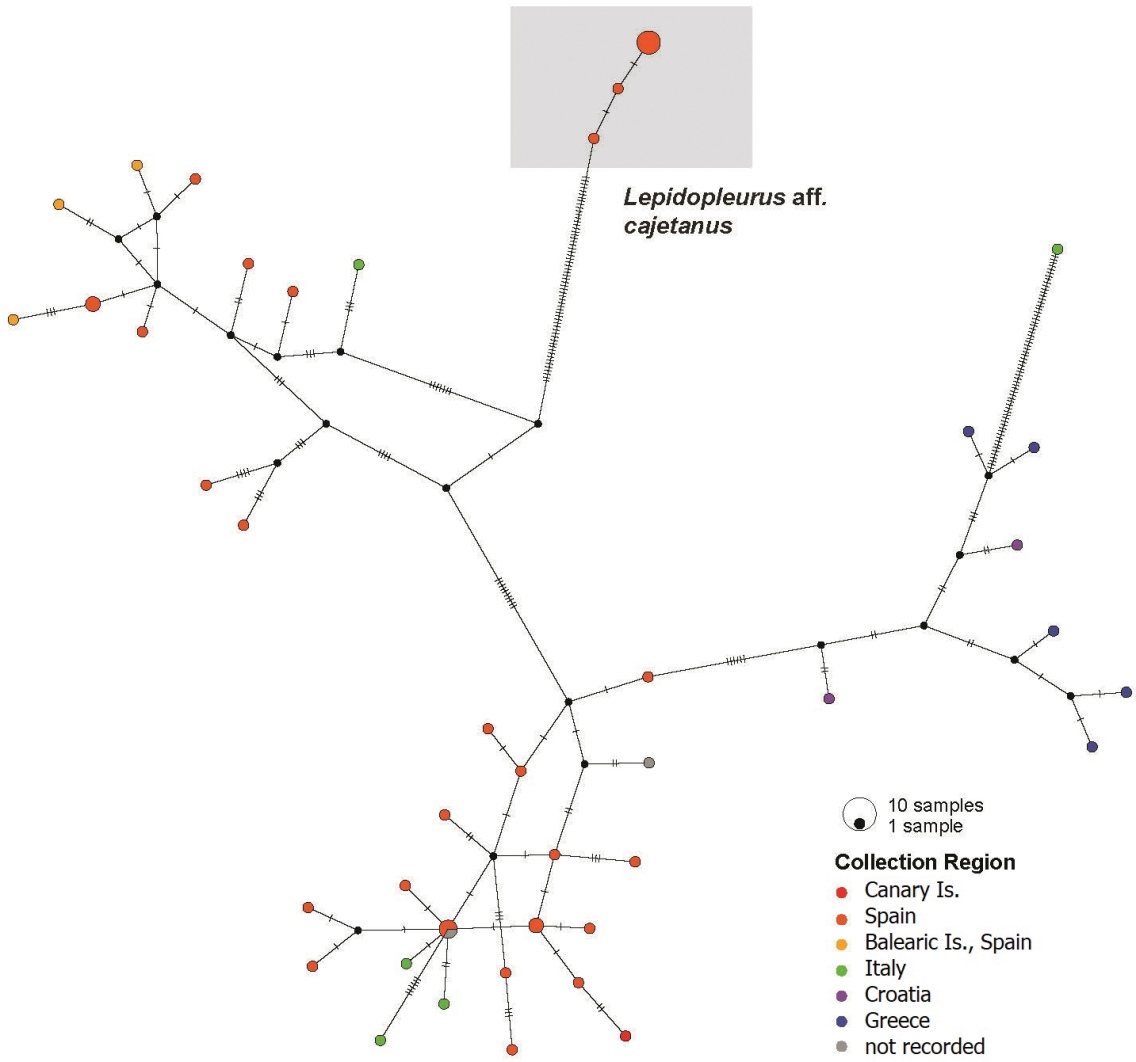
COI haplotype (TCS) network showing the relationships of *L.cajetanus* specimens. Circle size is proportional to the observed haplotype frequencies. Colours correspond to the geographic distribution as in Fig. 1 (see also Table 1).

Results from the ABGD based on the COI fragments recovered two distinct groups, plus a separate group represented by only one specimen (specimen 35, KJ500166). These two main groups correspond exactly to the two major clades of *Lepidopleuruscajetanus* recovered in the combined phylogenetic analysis (Fig. 1). The COI haplotype network reconstruction (Fig. 2) also resulted in two groups that also correspond to those identified by the barcode gap and phylogenetic analysis. By comparison of the outputs obtained from these analyses (ABGD and TCS haplotype networks) and considering the phylogenetic tree topology, it appears that the two groups form well resolved and distinct populations. As far as concerns specimen 35 (from Fernández et al. 2014) it nests within the primary *Lepidopleuruscajetanus* clade but is quite different from the others. The phylogenetic reconstruction for *L.cajetanus* shows a deep split, with two major clades supported by high (100%) posterior probability values. One of the clades is composed of individuals drawn from all the sampled populations, which we refer to as *Lepidopleuruscajetanus* s.s. The other clade, which we refer to as Lepidopleurusaff.cajetanus is formed by specimens from the eastern Iberian Peninsula (i.e., various localities of Girona, Alicante and Murcia including Cadaqués, Tossa del Mar, Xabia and Cabo de Palos), thus suggesting the presence of two genetically divergent lineages on the eastern Spanish coast. Fernández et al. (2014) sampled three specimens from the Balearic Islands (COI marker only, GenBank accession numbers KF052944–KF052946) which are part of the broader *Lepidopleuruscajetanus* s.s. lineage. Since both clades co-occur on the eastern Spanish mainland coast, hypothetically, we cannot exclude the possibility that L.aff.cajetanus may be present also in the Balearic Islands.

Comparing these two clades nominally comprising *Lepidopleuruscajetanus*, it appears that the L.aff.cajetanus clade has a much more limited genetic variability compared to the larger, more broadly distributed clade. The pairwise distances of COI fragments for the larger clade had a maximum separation of 8.3% (or up to 20% including specimen 35) and an average distance of 3.6%; the maximum distance between members of the L.aff.cajetanus clade was 0.49% with an average of 0.22%. This is reflected in the smaller distances and smaller number of haplotypes among the L.aff.cajetanus clade specimens (Fig. 2). It may be an artefact of comparative sample numbers for the COI fragment, with only seven specimens of the L.aff.cajetanus clade compared to 43 from *Lepidopleuruscajetanus* s.s., but the observed differences might indicate biological separation of the two lineages. The two clades are separated by a mean distance of 17.8%, which is similar to the value of 15.7% used as part of the description to separate *Leptochitoncascadiensis* from *Leptochitonrugatus* (Sigwart and Chen 2018).

Our results confirm that the population genetic structure of *Lepidopleuruscajetanus* based on the COI barcode marker is characterized by a high number of private haplotypes, and high genetic divergence between haplotypes and between clades, extending the pattern first identified by Fernández et al. (2014). However, with the addition of a broader geographical sampling, it seems that the “chaotic patchiness” nonetheless divides into two discrete clades, and further to some larger biogeographic patterns. The combined phylogenetic reconstruction shows one clade of specimens from Greece and Croatia, and two groupings of specimens from Italy and Spain, within the *Lepidopleuruscajetanus* s.s. clade. We suspect that the *Lepidopleuruscajetanus* s.s. clade and the L.aff.cajetanus clade might represent two distinct lineages, where *Lepidopleuruscajetanus* s.s. contains substantially more genetic diversity, at least in the COI marker, and the L.aff.cajetanus clade is more constrained. Whether L.aff.cajetanus could be interpreted as a possible (criptic?) species is impossible to say, as genetic data alone are not sufficient to draw any definitive conclusions. In fact, further morphological examination of Spanish specimens is certainly required to re-examine potential diagnostic characters, and to obtain additional independent sources of comparative data. Unfortunately, all of the sequence data corresponding to the L.aff.cajetanus clade came from prior work; we have not examined specimens known to be from the L.aff.cajetanus clade in the present work. In fact, new materials that we sequenced from Aguilas, Murcia, Spain, are also part of the *Lepidopleuruscajetanus* s.s. clade.

The fossil valves of *Lepidopleuruscajetanus* sensu lato show remarkable variations, e.g. in the sculpture of the lateral areas of the intermediate valves (with the starting point of the concentric ribs neighbouring the lateral margin and not near the apex, as in normal valves, and consequently with a different frontal view; compare Dell’Angelo et al. 2013: pl. 1 figs B-C and D-E), in the position of the mucro in the tail valves [almost central in juvenile specimens but moves posterior (even to the end of the valve) as individuals grew older, as well described and illustrated by Laghi (1977: fig. 3a-b) and Dell’Angelo et al. (2013: pl. 1, figs F-G)], and in the sculpture of the central area of the intermediate valves and the antemucronal area of the tail valve [normally with longitudinal and parallel chains of granules, somewhat branching or anastomosing, very irregular, transversally intersected by thinner cords that give a pitted appearance (see Dell’Angelo et al. 2015: pl. 1, figs 4–11)]. Future studies of material of living *Lepidopleuruscajetanus* s.l. from the eastern Spanish mainland coast (and Balearic Is.) should focus on these shell characteristics, to determine whether the two lineages can be diagnosed morphologically, and also how they compare to the extensive fossil record.

It is now well known that standard barcode markers such as COI show some variability within and among species (e.g., Sigwart and Garbett 2018), and it is not appropriate to use an a priori distance cut-off to distinguish species. Taking into account the limitations of the current study (reliance of mt-DNA only) and that species status is best assessed in light of an integrative, total evidence approach, caution is required in interpreting the L.aff.cajetanus clade until a morphological diagnosis is available. However, our results seem to suggest the presence of (at least) two genetic lineages within *L.cajetanus* that will need to be adequately investigated in future studies including also additional (nuclear) markers and/or anatomy to arrive at systematically more robust conclusions. Importantly, this is a species (or species complex) with a very good fossil record and representing greater disparity than the living lineages (Dell’Angelo et al. 2013, 2015, 2018a, 2018b). Although *Lepidopleuruscajetanus* s.s. has apparently high variability in these mitochondrial markers, we are cautious about making any inferences about phylogeographic patterns or potential for cryptic species or incipient speciation. These issues do require integrated evidence from the morphology of living and fossil populations, nonetheless this study indicates a novel genetic pattern in a common and phylogenetically important species.
